# Melanosis Coli

**DOI:** 10.31662/jmaj.2021-0031

**Published:** 2021-07-09

**Authors:** Akira Kuriyama

**Affiliations:** 1 Emergency and Critical Care Center, Kurashiki Central Hospital, Kurashiki, Japan

**Keywords:** melanosis, constipation, laxatives, anthraquinone

A 75-year-old man with a 23-year history of poorly controlled type 2 diabetes mellitus presented with chronic constipation. Colonoscopy showed heavily pigmented mucosa, resembling leopard skin, from the cecum through the ascending colon (Figure 1). The patient had been using senna glycoside for the last 7 years, which supported a diagnosis of melanosis coli. 

**Figure 1.**
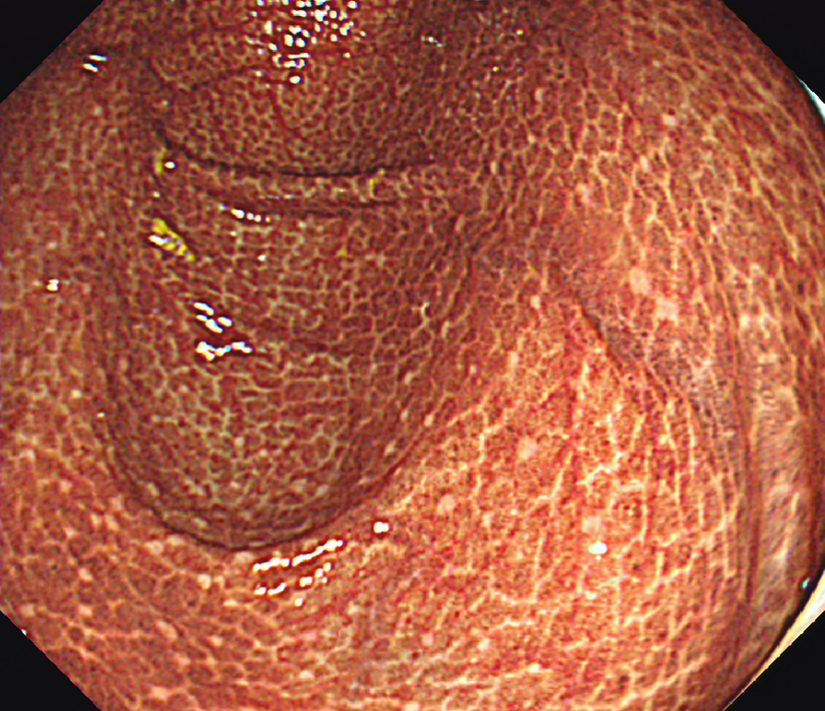
Colonoscopy showed heavily pigmented mucosa near the cecum.

A multicenter observational study with patients who had undergone colonoscopy suggested that the prevalence of melanosis coli is approximately 1.8% ^(1)^. Melanosis coli is associated with the chronic use of laxatives, particularly those containing anthraquinones, such as senna, rhubarb, and cascara. It can develop within a few months of using anthraquinone-containing laxatives. Anthraquinones cause direct injury to and apoptosis of the colonic epithelial cells, resulting in lipofuscin deposition in the macrophages of the lamina propria ^(2)^, which is visible as a dark pigment. Melanosis coli disappears with the discontinuation of the drug.

Although there is no known association between melanosis coli and colorectal cancer ^(3), (4)^, melanosis coli may be associated with a higher incidence and number of colonic non-adenoma polyps and low-grade adenomas ^(1), (5)^. Thus, follow-up colonoscopy should be considered in patients with melanosis coli.

## Article Information

### Conflicts of Interest

None

### Author Contributions

AK took care of the patient, wrote the manuscript, and submitted the current article.

### Informed Consent

Written informed consent was obtained from the patient to publish this case report including the accompanying images.
